# Educational and Psychological Support Combined with Minimally Invasive Surgical Technique Reduces Perioperative Depression and Anxiety in Patients with Bladder Cancer Undergoing Radical Cystectomy

**DOI:** 10.3390/ijerph182413071

**Published:** 2021-12-11

**Authors:** Artur Lemiński, Krystian Kaczmarek, Aleksandra Bańcarz, Alicja Zakrzewska, Bartosz Małkiewicz, Marcin Słojewski

**Affiliations:** 1 Department of Urology and Urological Oncology, Pomeranian Medical University, 71-899 Szczecin, Poland; k.kaczmarek.md@gmail.com (K.K.); mslojewski@gmail.com (M.S.); 2 Department of Psychiatry, Pomeranian Medical University, 71-457 Szczecin, Poland; bancarz.aleksandra@gmail.com; 3 Department of Radiology, Independent Public Provincial Hospital, 71-455 Szczecin, Poland; alicjazak@outlook.com; 4 University Center of Excellence in Urology, Department of Minimally Invasive and Robotic Urology, Wroclaw Medical University, 50-556 Wroclaw, Poland; bartosz.malkiewicz@umed.wroc.pl

**Keywords:** bladder cancer, cystectomy, psychological distress, anxiety, depression, support group

## Abstract

Radical cystectomy (RC) for muscle-invasive bladder cancer (MIBC) is an extensive and morbid operation, often associated with permanent alteration of body image and disability. Combined with the aggressive malignant potential of MIBC and considerable risk of complications, it poses a serious threat to the psychological well-being of patients. Educational deficiencies causing uncertainty and confusion aggravate surgery-related anxiety and may lead to depression along with further social disability. We conceived a preoperative supportive program named “Cystocare” held by urologists, psychologists, stoma therapists and cancer survivors to facilitate patients’ adaptation and coping. We aimed to evaluate whether participation in Cystocare meetings would alleviate emotional distress in patients undergoing RC. We included 95 consecutive patients who filled Hospital Anxiety and Depression Score questionnaires before RC and on discharge. The intervention arm (A) comprised 32 Cystocare participants. The remaining 63 patients who received standard preparation constituted the control arm (B). Whilst there were no differences in median anxiety and depression scores preoperatively, in postoperative measurement, the intervention arm showed a lower median depression score than controls: 3 vs. 8 points, *p* = 0.015. On multivariate analysis we confirmed lower risk of postoperative depression in Cystocare participants: OR = 0.215 (95%CI: 0.066–0.699), *p* = 0.011, along with lower odds of preoperative anxiety in patients undergoing laparoscopic RC: OR = 0.365 (95%CI: 0.136–0.978), *p* = 0.045, and higher risk of prolonged hospital stay in patients experiencing postoperative anxiety OR = 17.114 (95%CI: 1.283–228.234) *p* = 0.032. Preoperative educational and supportive intervention complements laparoscopic RC in the alleviation of surgery-related anxiety and depression. The support group meetings provide an attractive and cost-effective opportunity to moderate emotional response in patients undergoing RC, and as such, deserve widespread adoption.

## 1. Introduction

Radical cystectomy (RC) with pelvic lymphadenectomy remains the mainstay of surgical treatment for muscle-invasive bladder cancer (MIBC) [1]. It is an extensive surgical procedure, and regardless of the surgical technique used, it is associated with substantial morbidity and a high burden of complications [2]. The majority of patients would still receive an incontinent urinary reconstruction, which implicates the creation of a unilateral or bilateral urostomy, leading to a permanent depreciation of body image and disability. Radical cystectomy is a life-transforming surgery affecting numerous aspects of patients’ functioning, which include, but are not limited to, self-care and stoma management, everyday coping, sexuality and psychological well-being. All these factors combined with informational and supportive deficiencies not infrequently encountered in everyday practice of urological departments, may contribute to significant psychological distress of patients with MIBC scheduled to undergo RC [3,4]. 

We sought to evaluate whether a preoperative supportive and educational intervention within a support group program named ‘Cystocare’ would lead to an improvement in patients’ mental well-being within a perioperative period.

## 2. Materials and Methods

Our study was conducted amongst patients undergoing RC in the Department of Urology of the Pomeranian Medical University, Szczecin, Poland. The protocol was designed with respect to the regulations set forth by the Declaration of Helsinki. The study was exempt from ethical approval by the Institutional Review Board (Bioethical Committee) of the Pomeranian Medical University, as the study intervention had no potential for harm, and data gathering was conducted as a part of routine care (protocol no: KB-0012/05/01/2021/Z). Patients scheduled for surgery were invited to participate in Cystocare meetings together with their families and caregivers. The meetings were held on a monthly basis since April 2017, run by a team of urologists, stoma therapists, clinical psychologists and educators—patients who underwent RC in the past and volunteered to share their experience. Each meeting consisted of two parts. The first educational part included a short lecture on RC, types of urinary reconstruction, necessary preparation for the surgery, and a day-to-day description of a typical hospital stay of a cystectomy patient. Patients were also familiarized with their part of Enhanced Recovery After Surgery (ERAS) protocol procedures, with a strong emphasis placed on diet and a pre-habilitation program involving a gradual increase in aerobic physical activity, cessation of smoking and alcohol, along with medical optimization of their comorbidities. The second psychotherapeutic and supportive part of the meeting included individual consultations with a clinical psychologist, stoma therapist and discussions with educators on various aspects of everyday life after RC. 

From the study population, we excluded patients with significant cognitive impairment and patients with a previous diagnosis of anxiety or depression and history of psychiatric treatment. Consequently, we prospectively enrolled 95 patients in the study cohort, which was divided into two arms: patients who participated in Cystocare program meetings (arm A) and a standard preparation arm (arm B), which included patients who did not respond to invitation or refused to participate and were prepared for RC in a traditional way. The latter included distribution of information brochure on RC and a preoperative meeting with a surgeon and stoma therapist. Intensities of psychological distress symptoms were measured using the Hospital Anxiety and Depression Scale (HADS) questionnaire in both study arms. The questionnaire was used twice for each participant— a baseline assessment was made on the day before surgery, and a postoperative one on the day of discharge. All patients were informed and provided written consent before participation in procedures related to the study. 

The HADS questionnaire allowed for separate measurements of anxiety and depression intensity, and these were compared between study arms A and B. We used the recommended cut-off value of eight points for each of the HADS subdomains to identify patients at risk for anxiety and depression, respectively [5]. We also sought to evaluate other factors influencing patients’ mental well-being and therefore performed a multivariate analysis of additional clinical features potentially influencing their emotional comfort. These included: age, sex, surgical access (open vs. laparoscopic), length of hospital stay (LOS: ≤7 vs. >7 days), incidence of severe (grade 3–4) complications and type of urinary diversion. Data were checked for internal consistency. Testing for normality of distribution was performed objectively using a Shapiro-Wilk test and subjectively by observing the histograms and box plots. In cases where the assumption of normal distribution was not met, a Box-Cox transformation was applied (Table S1). Homogeneity of variances was checked by the Levene test. Descriptive statistics, including mean ± standard deviation (SD) and median (interquartile range—IQR), were provided for normally distributed and skewed data, respectively. Single variables were compared using an independent *t*-test for parametric variables and a Mann-Whitney U-test for non-parametric variables. The multivariate analysis was performed with generalized nonlinear models, i.e., the logistic regression. Predictive values of logistic regression models were evaluated with the receiver-operator curve (ROC), and the area under curve (AUC) calculation was performed for each model (Table S2). We considered *p*-value < 0.05 statistically significant, and all *p*-values were two-sided. All tests were performed with Statistica software, version 13.5 (TIBCO Software Inc., Palo Alto, CA, USA). 

## 3. Results

Ninety-five consecutive patients who correctly filled and returned HADS questionnaires constituted the study group. In a cut-off level analysis, 43 (45.3%) and 37 (38.9%) patients had preoperative anxiety and depression, respectively. After RC, the anxiety and depression rates have not decreased significantly. Postoperative anxiety was noted in 37 patients (38.9%; *p* = 0.185), whereas 36 patients were at risk of depression (37.9% *p* = 0.444). Thirty-two (33.6%) patients responded to the invitation and agreed to participate in the Cystocare meetings. Younger patients were more likely to join the program (mean age arm A: 64.7 SD 8.25; arm B: 68.8 SD 7.87; *p* = 0.021). There were no further differences in demographic and clinical features of patients between both study arms (Table 1). 

The median preoperative anxiety and depression scores were comparable in both arms: HADS baseline - Arm A anxiety: 7 points, Arm B anxiety: 8 points, *p* = 0.096; Arm A depression: 5 points, Arm B depression: 6.5 points, *p* = 0.302. Furthermore, in multivariate analysis, attending the Cystocare meeting had no influence on the preoperative level of anxiety and depression. Nonetheless, we found a lower risk for borderline/abnormal preoperative anxiety in patients planned for laparoscopic RC, compared to those scheduled for an open operation: OR = 0.365 (95%CI: 0.136–0.978), *p* = 0.045 (Table 2).

The median postoperative anxiety score was similar in both study arms: Arm A: 6 points, Arm B: 8 points, *p* = 0.127. However, there was a significantly lower median postoperative depression score in patients from Arm A: 3 points, compared to patients from Arm B: 8 points, *p* = 0.015. Moreover, in a multivariate analysis of postoperative data, we found that there was a lower risk of borderline/abnormal depression score in patients who participated in Cystocare meetings (Arm A) OR = 0.215 (95%CI: 0.066–0.699) *p* = 0.011 (Table 3).

In an additional evaluation of factors associated with prolonged length of hospital stay, we found it was strongly associated with an abnormal postoperative anxiety score OR = 17.114 (95%CI: 1.283–228.234) *p* = 0.032 and with the type of surgical approach (laparoscopic vs. open RC), favoring the laparoscopic technique OR = 0.153 (95%CI: 0.034–0.688) *p* = 0.014 (Table 4).

## 4. Discussion

The psychological distress symptoms, specifically depression and anxiety, remain an inherent part of oncological surgery. This is particularly true for RC patients in whom the prevalence of psychological distress is higher than in those affected with other cancers [6]. The emotional response may be a function of different factors such as diagnosis of an aggressive tumor with uncertain long-term prognosis, large extent of the imminent surgical procedure and the need for an alternative urinary diversion, frequently leading to a deterioration of body image and permanent disability. Bearing in mind the complexity of the emotional response, preoperative support and psychological preparation to RC is likely to play a substantial role, particularly given that patient reported mental health was independently associated with incidence of high-grade complications after cystectomy [7].

Most studies investigating emotional response to surgery have demonstrated an elevated risk of anxiety and depression before surgery, followed by a significant decrease thereafter. This phenomenon was described amongst patients subjected to emergency, as well as major elective, surgeries including RC [6,8]. In our study, more than one-third of patients scheduled for RC had anxiety and depression symptoms within the mild to severe range (HADS score ≥ 8). Interestingly, the overall proportion of patients at risk did not decrease significantly on the day of discharge. This is in line with findings from Benner et al., who observed an increase in depression symptoms during follow-up after RC. In the aforementioned study, the pre-cystectomy average HADS scores for anxiety and depression were within the normal range, whereas at 4 and 6 months after RC, they increased to borderline abnormal levels [9]. This may indicate that in certain groups of patients with MIBC treated with RC, distress may continue to accumulate after surgery, a phenomenon possibly related to urinary diversion type and supportive undertakings. Taking the above into account, establishing mechanisms to preemptively moderate the emotional response of cystectomy patients seems a very attractive pathway.

There is now a general understanding that educational and psychological preparation for surgery is advantageous for most patients; however, there is a paucity of evidence for patients who were scheduled for elective RC [10]. The effects of psychological preparation have mostly been measured after non-urological surgeries [11,12]. Moreover, the influence of preoperative psychological preparation was usually measured 3 months or later after surgery [12]. These outcomes may, to a greater extent, represent preparation for the long-term sequelae of the procedure rather than preparation to handle procedure-related stress itself. Taking this into account, in our study, we restricted the assessment of anxiety and depression to the immediate perioperative period. Consequently, we chose the Hospital Anxiety and Depression Scale, designed primarily for hospital use and, despite its validation in a variety of clinical settings, we decided to use it only for inpatients [13]. This study design enabled us to investigate a direct association between preoperative supportive and psychological intervention and early emotional response to RC. 

It remains undetermined which preparation techniques would bring the most benefit to a population of patients with MIBC undergoing RC. To date, several forms of preoperative supportive and educational interventions have been proposed, and some have proven effective in improving physical performance, emotional functioning and self-caring of patients after cystectomy [14,15]. Our study revealed a significant reduction in postoperative depression within a subgroup of patients who joined Cystocare meetings and followed the customized pre-habilitation program during preparation for surgery. Effects of psychotherapy on depressive symptoms in cancer patients have been summarized in a recent systematic review, which demonstrated that cognitive behavioral therapy (CBT) brings the best outcome [16]. However, this review included only incurable cancer patients, and the number of those with BC was limited. The effectiveness of CBT after surgery for early-stage cancer patients was reported by Stagl et al. Women with stage 0 to IIIb breast cancer were recruited after surgery and randomized to cognitive-behavioral stress management or control group. Participants assigned to the intervention group reported significantly lower depressive symptoms OR 0.63 (95%CI: 0.56–0.70) [12]. The abovementioned findings are obviously difficult to translate into perioperative settings and associated depressive symptoms of a cystectomy patient. We believe our study contributes to the scarce evidence on the effectiveness of preemptive supportive and psychotherapeutic intervention in alleviating perioperative psychological distress symptoms amongst cystectomy patients.

Our study also showed a beneficial influence of minimally invasive surgical techniques on preoperative anxiety. Patients due to undergo a laparoscopic RC suffered significantly less anxiety than those who were planned for an open procedure. Interestingly, conflicting results were presented by a recent comparison of laparoscopic and open cholecystectomy. Patients who underwent laparoscopic cholecystectomy more frequently presented moderate to high levels of anxiety preoperatively [17]. These surprising results may suggest difficulties in patients’ adaptation to rapid technical developments in surgery. Some patients may find it difficult to believe that surgery can safely be accomplished through a small incision, a phenomenon that emphasizes the importance of thorough patient information. Therefore, patients who are operated in high-volume centers with extensive experience in laparoscopic surgery may experience lower anxiety before minimally invasive surgery. This may be one of the plausible explanations of our results. Our department is a pioneer and one of the highest volume centers for laparoscopic RC in Poland and closely follows all developments in this field. Nevertheless, to our knowledge, our study is the first to demonstrate such benefits of a laparoscopic approach to RC. 

Our study also revealed the association between abnormal levels of postoperative anxiety and hospital stay longer than a week, even in absence of high-grade complications. This finding has some plausible explanations. Patients with an increased anxiety level are more often reluctant to leave the hospital earlier, as they may worry about being challenged with the extent of disability and a new everyday reality at home [18,19]. At the same time, a prolonged hospital stay may represent a source of anxiety itself, when patients are becoming increasingly concerned about the postoperative course not going exactly according to plan.

We need to acknowledge certain limitations of this study. The COVID-19 pandemic and subsequent epidemiological measures suspended the Cystocare meetings together with study recruitment in early 2020. Therefore, the analysis in this preliminary report has been performed on a limited patient sample, and the Cystocare arm was smaller than the controls. These conditions have restricted some of the statistical analyses, including predictive values of some logistic regression models, i.e., preoperative feelings of anxiety and depression. Moreover, none of the patients from our cohort received an orthotopic bladder substitution. Patients from the intervention arm were also significantly younger than those who received standard preparation, which may represent their more proactive approach to treatment and more positive attitude towards impending surgery. Nonetheless, we found that patient age was not independently associated with any of the HADS domain sub-scores.

## 5. Conclusions

Preoperative educational and supportive intervention within a support group program improves patients’ adjustment to sequelae of radical cystectomy, resulting in a decrease of depression intensity on discharge. Minimally invasive surgical technique is associated with lower intensity of preoperative anxiety, and allowing shorter hospital stay, contributes to a decrease in the proportion of patients experiencing intensified postoperative anxiety. Support group meetings provide an attractive and cost-effective opportunity to moderate emotional response in patients undergoing RC, and as such, deserve widespread adoption.

## Figures and Tables

**Table 1:** Characteristics of the study group.

Variable	Cystocare Meeting		
	No	Yes	*p*
*n*	63	32	
**Age**			0.021 *
mean	68.82	64.75	
SD	7.87	8.25	
**Gender**			0.114 **
female	14	12	
male	49	20	
**Marital status**			0.725 **
single	12	13	
married	41	19	
**Length of stay**			0.921 **
median (days)	8	8	
**Surgery**			0.055 **
open	24	6	
laparoscopic	39	26	
**Complications grade 3/4**			0.975 **
no	53	27	
yes	10	5	
**Urinary diversion**			0.279 **
ureterostomy	33	13	
ileal conduit	29	19	

SD: standard deviation, * independent *t*-test; ** Mann-Whitney U-test.

**Table 2:** Analysis of clinical factors associated with borderline or abnormal preoperative feelings of anxiety and depression in HADS (generalized nonlinear model, multivariate logistic regression).

		Anxiety				Depression			
Variables		OR	95%CI	95%CI	*p*	OR	95%CI	95%CI	*p*
Age									
≤65		Ref.				Ref.			
>65		1.150	0.447	2.959	0.772	1.038	0.408	2.642	0.937
**Gender**									
male		Ref.				Ref.			
female		1.585	0.574	4.380	0.375	1.087	0.397	2.976	0.871
**Marital status**									
single		Ref.							
married		0.652	0.261	1.632	0.361	0.579	0.236	1.420	0.232
**Surgical approach**									
open		Ref.				Ref.			
laparoscopic		0.365	0.136	0.978	0.045	0.568	0.218	1.478	0.246
**Preoperative meeting**									
no		Ref.				Ref.			
yes		0.532	0.200	1.417	0.207	0.635	0.238	1.692	0.364

OR: odds ratio; CI: confidence interval.

**Table 3:** Analysis of clinical factors associated with borderline or abnormal postoperative feelings of anxiety and depression in HADS (generalized nonlinear model, multivariate logistic regression).

		Anxiety				Depression			
Variables		OR	95%CI	95%CI	*p*	OR	95%CI	95%CI	*p*
**Age**									
≤65		Ref.				Ref.			
>65		0.769	0.274	2.161	0.618	1.304	0.455	3.737	0.621
**Gender**									
male		Ref.				Ref.			
female		1.772	0.598	5.246	0.302	1.300	0.430	3.931	0.642
**Marital status**		Ref.				Ref.			
single									
married		1.234	0.462	3.292	0.675	0.669	0.248	1.802	0.427
**Length of stay**									
≤7		Ref.				Ref.			
>7		2.857	0.953	8.568	0.061	2.333	0.773	7.043	0.133
**Surgical approach**									
open		Ref.				Ref.			
laparoscopic		1.094	0.367	3.261	0.872	1.143	0.378	3.460	0.813
**Preoperative meeting**									
no		Ref.				Ref.			
yes		0.353	0.114	1.087	0.069	0.215	0.066	0.699	0.011
**Urinary diversion**									
ureterostomy		Ref.				Ref.			
ileal conduit		1.568	0.562	4.378	0.391	1.384	0.485	3.950	0.543
**Complications grade**									
None/<2		Ref.				Ref.			
>2		0.701	0.194	2.540	0.589	0.696	0.192	2.521	0.581

OR: odds ratio; CI: confidence interval.

**Table 4:** Multivariate analysis of clinical factors associated with hospital stay ≥7 days (generalized nonlinear model, multivariate logistic regression).

Variable		OR	95%CI	95%CI	*p*
Age					
≤65		Ref.			
>65		0.521	0.157	1.736	0.289
**Gender**					
male		Ref.			
female		1.036	0.280	3.835	0.957
**Marital status**					
single		Ref.			
married		0.439	0.127	1.516	0.193
**Operation approach**					
open		Ref.			
laparoscopic		0.153	0.034	0.688	0.014
**Preoperative meeting**					
no		Ref.			
yes		3.140	0.808	12.205	0.099
**Postoperative feeling of anxiety**					
normal		Ref.			
borderline		1.643	0.381	7.090	0.506
abnormal		17.114	1.283	228.234	0.032
**Postoperative feeling of depression**					
normal		Ref.			
borderline		1.091	0.184	6.480	0.924
abnormal		0.952	0.162	5.592	0.957
**Urinary diversion**					
ureterocutaneostomy		Ref.			
ileal conduit		2.494	0.730	8.525	0.145
**Complications**					
≤2		Ref.			
≥3		1.091	0.255	4.670	0.907

OR: odds ratio; CI: confidence interval.

## Data Availability

https://osf.io/gcdt3 (accessed on 8 December 2021).

## Supplementary Materials

The following are available online at https://www.mdpi.com/article/10.3390/ijerph182413071/s1. Table S1. Age sampling distributions before and after Box-Cox transformation. Table S2. Logistic regression models—predictive value evaluations.
