# Supplementary material for: Educational and Psychological Support Combined with Minimally Invasive Surgical Technique Reduces Perioperative Depression and Anxiety in Patients with Bladder Cancer Undergoing Radical Cystectomy
Source: Int J Environ Res Public Health. 2021 Dec 11;18(24):13071. doi: 10.3390/ijerph182413071 (PMC8701127; doi:10.3390/ijerph182413071)
Supplement: Supplementary file 1 [file ijerph-18-13071-s001.zip › Table S1.pdf]

Sampling distribution before Box Cox transformation.

	N	mean	median	mode	min.	max.	lower quantile	upper quantile	variance	standard deviation	coefficient of variation	skewness	kurtosis
All	95	67,45	67,00	66,00000	41,00	89,00	63,00	73,00	67,10	8,19	12,14	-0,34	0,96
Arm A	32	64,75	66,00	66,00000	41,00	76,00	61,50	69,50	68,13	8,25	12,75	-0,93	1,16
Arm B	63	68,83	68,00	66,00000	47,00	89,00	64,00	74,00	61,99	7,87	11,44	-0,01	0,45

Sampling distribution after Box Cox transformation.

	N	mean	median	mode	min.	max.	lower quantile	upper quantile	variance	standard deviation	coefficient of variation	skewness	kurtosis
All	95	893,47	874,72	852,0697	371,22	1435,37	785,65	1015,89	34174,72	184,86	20,69	0,03	0,65
Arm A	32	832,62	852,07	852,0697	371,22	1089,82	753,33	932,48	30895,28	175,77	21,11	-0,62	0,45
Arm B	63	924,38	897,62	852,0697	471,18	1435,37	807,53	1040,29	33484,05	182,99	19,80	0,29	0,40
