# Supplementary material for: Educational and Psychological Support Combined with Minimally Invasive Surgical Technique Reduces Perioperative Depression and Anxiety in Patients with Bladder Cancer Undergoing Radical Cystectomy
Source: Int J Environ Res Public Health. 2021 Dec 11;18(24):13071. doi: 10.3390/ijerph182413071 (PMC8701127; doi:10.3390/ijerph182413071)
Supplement: Supplementary file 1 [file ijerph-18-13071-s001.zip › Table S2.pdf]

Analysis of clinical factors associated with borderline or abnormal preoperative feeling of anxiety in HADS

| | coefficient | standard error coefficient | Wald chi-square | lower 95% CI coefficient | upper 95% CI coefficient | *p* | OR | lower 95% CI OR | upper 95% CI OR |
|---|---|---|---|---|---|---|---|---|---|
| Intercept | 0,877 | 0,579 | 2,292 | -0,258 | 2,013 | 0,130 | 2,404 | 0,772 | 7,482 |
| Cystocare meeting (no vs yes) | -0,631 | 0,500 | 1,594 | -1,610 | 0,348 | 0,207 | 0,532 | 0,200 | 1,417 |
| Marital status (single vs married) | -0,428 | 0,468 | 0,835 | -1,345 | 0,490 | 0,361 | 0,652 | 0,261 | 1,632 |
| Gender (male vs female) | 0,461 | 0,519 | 0,789 | -0,556 | 1,477 | 0,375 | 1,585 | 0,574 | 4,380 |
| Age (≤65 vs > 65) | 0,140 | 0,482 | 0,084 | -0,805 | 1,085 | 0,772 | 1,150 | 0,447 | 2,959 |
| Surgical approach (open vs laparoscopic) | -1,008 | 0,503 | 4,015 | -1,994 | -0,022 | 0,045 | 0,365 | 0,136 | 0,978 |
| AUC | 0.688 | | | | | | | | |
| AUC error | 0.0569 | | | | | | | | |
| Hosmer Lemeshow test ( p value) | 0.969 | | | | | | | | |

Analysis of clinical factors associated with borderline or abnormal preoperative feeling of depression in HADS

| | coefficient | standard error coefficient | Wald chi-square | lower 95% CI coefficient | upper 95% CI coefficient | *p* | OR | lower 95% CI OR | upper 95% CI OR |
|---|---|---|---|---|---|---|---|---|---|
| Intercept | 0,455 | 0,559 | 0,662 | -0,641 | 1,550 | 0,416 | 1,575 | 0,527 | 4,710 |
| Cystocare meeting (no vs yes) | -0,454 | 0,500 | 0,825 | -1,435 | 0,526 | 0,364 | 0,635 | 0,238 | 1,692 |
| Marital status (single vs married) | -0,547 | 0,458 | 1,428 | -1,445 | 0,350 | 0,232 | 0,579 | 0,236 | 1,420 |
| Gender (male vs female) | 0,083 | 0,514 | 0,026 | -0,924 | 1,091 | 0,871 | 1,087 | 0,397 | 2,976 |
| Age (≤65 vs > 65) | 0,038 | 0,476 | 0,006 | -0,896 | 0,972 | 0,937 | 1,038 | 0,408 | 2,642 |
| Surgical approach (open vs laparoscopic) | -0,566 | 0,488 | 1,346 | -1,523 | 0,390 | 0,246 | 0,568 | 0,218 | 1,478 |
| AUC | 0.629 | | | | | | | | |
| AUC error | 0.0618 | | | | | | | | |
| Hosmer Lemeshow test ( p value) | 0.199 | | | | | | | | |

Analysis of clinical factors associated with borderline or abnormal postoperative feeling of anxietyin in HADS

| | coefficient | standard error coefficient | Wald chi-square | lower 95% CI coefficient | upper 95% CI coefficient | *p* | OR | lower 95% CI OR | upper 95% CI OR |
|---|---|---|---|---|---|---|---|---|---|
| Intercept | -0,814 | 0,785 | 1,074 | -2,352 | 0,725 | 0,300 | 0,443 | 0,095 | 2,065 |
| Cystocare meeting (no vs yes) | -1,044 | 0,575 | 3,295 | -2,171 | 0,083 | 0,069 | 0,352 | 0,114 | 1,087 |
| Marital status (single vs married) | 0,210 | 0,501 | 0,176 | -0,772 | 1,192 | 0,675 | 1,234 | 0,462 | 3,292 |
| Hospital stay (≤7 vs >7) | 1,050 | 0,560 | 3,510 | -0,048 | 2,148 | 0,061 | 2,857 | 0,953 | 8,568 |
| Gender (male vs female) | 0,572 | 0,554 | 1,067 | -0,513 | 1,657 | 0,302 | 1,772 | 0,598 | 5,246 |
| Age (≤65 vs > 65) | -0,263 | 0,527 | 0,249 | -1,296 | 0,771 | 0,618 | 0,769 | 0,274 | 2,161 |
| Surgical approach (open vs laparoscopic) | 0,090 | 0,557 | 0,026 | -1,003 | 1,182 | 0,872 | 1,094 | 0,367 | 3,261 |
| Urinary diversion (ureterocutaneostomy vs ileal conduit) | 0,450 | 0,524 | 0,737 | -0,577 | 1,477 | 0,391 | 1,568 | 0,562 | 4,378 |
| Complications grade (≤2 vs >2) | -0,355 | 0,657 | 0,292 | -1,642 | 0,932 | 0,589 | 0,701 | 0,194 | 2,540 |
| AUC | 0.709 | | | | | | | | |
| AUC error | 0.0597 | | | | | | | | |
| Hosmer Lemeshow test ( p value) | 0.741 | | | | | | | | |

Analysis of clinical factors associated with borderline or abnormal postoperative feeling of depression in HADS

| | coefficient | standard error coefficient | Wald chi-square | lower 95% CI coefficient | upper 95% CI coefficient | *p* | OR | lower 95% CI OR | upper 95% CI OR |
|---|---|---|---|---|---|---|---|---|---|
| Intercept | -0,479 | 0,795 | 0,363 | -2,038 | 1,079 | 0,547 | 0,619 | 0,130 | 2,943 |
| Cystocare meeting (no vs yes) | -1,536 | 0,601 | 6,532 | -2,714 | -0,358 | 0,011 | 0,215 | 0,066 | 0,699 |
| Marital status (single vs married) | -0,402 | 0,506 | 0,632 | -1,393 | 0,589 | 0,427 | 0,669 | 0,248 | 1,802 |
| Hospital stay (≤7 vs >7) | 0,847 | 0,564 | 2,257 | -0,258 | 1,952 | 0,133 | 2,333 | 0,773 | 7,043 |
| Gender (male vs female) | 0,262 | 0,565 | 0,216 | -0,844 | 1,369 | 0,642 | 1,300 | 0,430 | 3,931 |
| Age (≤65 vs > 65) | 0,265 | 0,537 | 0,244 | -0,787 | 1,318 | 0,621 | 1,304 | 0,455 | 3,737 |
| Surgical approach (open vs laparoscopic) | 0,134 | 0,565 | 0,056 | -0,974 | 1,241 | 0,813 | 1,143 | 0,378 | 3,460 |
| Urinary diversion (ureterocutaneostomy vs ileal conduit) | 0,325 | 0,535 | 0,369 | -0,723 | 1,374 | 0,543 | 1,384 | 0,485 | 3,950 |
| Complications grade (≤2 vs >2) | -0,363 | 0,657 | 0,305 | -1,651 | 0,925 | 0,581 | 0,696 | 0,192 | 2,521 |
| AUC | 0.712 | | | | | | | | |
| AUC error | 0.0586 | | | | | | | | |
| Hosmer Lemeshow test ( p value) | 0.656 | | | | | | | | |

Multivariate analysis of clinical factors associated with hospital stay ≥ 7 day

| | coefficient | standard error coefficient | Wald chi-square | lower 95% CI coefficient | upper 95% CI coefficient | *p* | OR | lower 95% CI OR | upper 95% CI OR |
|---|---|---|---|---|---|---|---|---|---|
| Intercept | 1,541 | 0,844 | 3,334 | -0,113 | 3,195 | 0,068 | 4,669 | 0,893 | 24,409 |
| Cystocare meeting (no vs yes) | 1,144 | 0,693 | 2,727 | -0,214 | 2,502 | 0,099 | 3,140 | 0,808 | 12,205 |
| Marital status (single vs married) | -0,822 | 0,632 | 1,693 | -2,061 | 0,416 | 0,193 | 0,439 | 0,127 | 1,516 |
| Gender (male vs female) | 0,036 | 0,668 | 0,003 | -1,273 | 1,344 | 0,957 | 1,036 | 0,280 | 3,835 |
| Age (≤65 vs > 65) | -0,651 | 0,614 | 1,126 | -1,854 | 0,551 | 0,289 | 0,521 | 0,157 | 1,736 |
| Surgical approach (open vs laparoscopic) | -1,880 | 0,768 | 5,991 | -3,385 | -0,375 | 0,014 | 0,153 | 0,034 | 0,688 |
| Urinary diversion (ureterocutaneostomy vs ileal conduit) | 0,914 | 0,627 | 2,124 | -0,315 | 2,143 | 0,145 | 2,494 | 0,730 | 8,525 |
| Complications grade (≤2 vs >2) | 0,087 | 0,742 | 0,014 | -1,368 | 1,541 | 0,907 | 1,091 | 0,255 | 4,670 |
| Postoperative depression | | | | | | | | | |
| normal vs borderline | 0,087 | 0,909 | 0,009 | -1,695 | 1,869 | 0,924 | 1,091 | 0,184 | 6,480 |
| normal vs abnormal | -0,049 | 0,903 | 0,003 | -1,820 | 1,721 | 0,957 | 0,952 | 0,162 | 5,592 |
| Postoperative anxiety | | | | | | | | | |
| normal vs borderline | 0,497 | 0,746 | 0,443 | -0,966 | 1,959 | 0,506 | 1,643 | 0,381 | 7,090 |
| normal vs abnormal | 2,840 | 1,322 | 4,617 | 0,249 | 5,430 | 0,032 | 17,114 | 1,283 | 228,234 |
| AUC | 0.812 | | | | | | | | |
| AUC error | 0.0488 | | | | | | | | |
| Hosmer Lemeshow test ( p value) | 0.596 | | | | | | | | |
